# Wide dynamic range and real-time reagent identification and imaging using multi-wavelength terahertz parametric generation and machine learning

**DOI:** 10.1038/s41598-023-40013-y

**Published:** 2023-08-07

**Authors:** Kosuke Murate, Sota Mine, Yuki Torii, Hyuga Inoue, Kodo Kawase

**Affiliations:** Department of Electronics, Graduate School of Engineering, Nagoya University, Furocho, Chikusa, Nagoya, 4648603 Japan

**Keywords:** Optical spectroscopy, Imaging and sensing

## Abstract

In this study, we propose a technique for identifying and imaging reagents through shielding over a wide dynamic range using a real-time terahertz (THz) spectroscopy system with multi-wavelength THz parametric generation/detection and machine learning. To quickly identify reagents through shielding, the spectral information of the “detection Stokes beam” is used for reagent recognition via machine learning. In general THz wave-based reagent identification, continuous spectra are acquired and analyzed quantitatively by post-processing. In actual applications, however, such as testing for illicit drugs in mail, the technology must be able to quickly identify reagents as opposed to quantifying the amount present. In multi-wavelength THz parametric generation/detection, THz spectral information can be measured instantly using a “multi-wavelength detection Stokes beam” and near-infrared (NIR) camera. Moreover, machine learning enables reagent identification in real-time and over a wide dynamic range. Furthermore, by plotting the identification results as pixel values, the spatial distribution of reagents can be imaged at high speed without the need for post-processing.

## Introduction

Because terahertz (THz) waves have both the fingerprint spectrum of reagents and material transparency, they are expected to be especially useful for the identification of hidden objects (e.g., testing for illicit drugs or explosives hidden in mail)^1–3^. However, since the transparency of THz waves is not very high, a real-time spectrometer^4,5^ with a high dynamic range is required. It is also essential that spectroscopic performance is unaffected by the scattering of THz waves by shielding.

Methods that have been proposed for real-time measurement include the use of a single-frequency source^6–8^, THz time-domain spectroscopy (THz-TDS)^9–11^, and multi-wavelength fast-switching injected-seeded THz parametric generation^12–16^.

Our research has focused on the development of THz spectrometers, mainly based on the injection-seeded THz parametric generator (is-TPG)^16,17^. Because the is-TPG is a wavelength-tunable source, the measurement time increases with the number of wavelengths involved. In addition, spectroscopic imaging requires several hours of measurement, as well as post-processing of the obtained images. Therefore, there is a need for one-shot spectroscopy and real-time identification that can significantly shorten the measurement time. We proposed a multi-wavelength generation/detection is-TPG system and successfully obtained spectra in one shot^15,16^; however, automatic identification in real-time has not yet been realized. Therefore, in this study, we applied machine learning^18^ for the identification of spectra obtained in one shot. The goal was to devise a practical system to rapidly identify reagents, even through thick shields with attenuation rates of − 60 dB. Furthermore, by using this system for spectroscopic imaging, the information in each pixel can be identified instantly, making it possible to determine the spatial distribution of reagents in a 40 × 40 mm^2^ area within a few tens of seconds.

## Results

**Figure 1:**
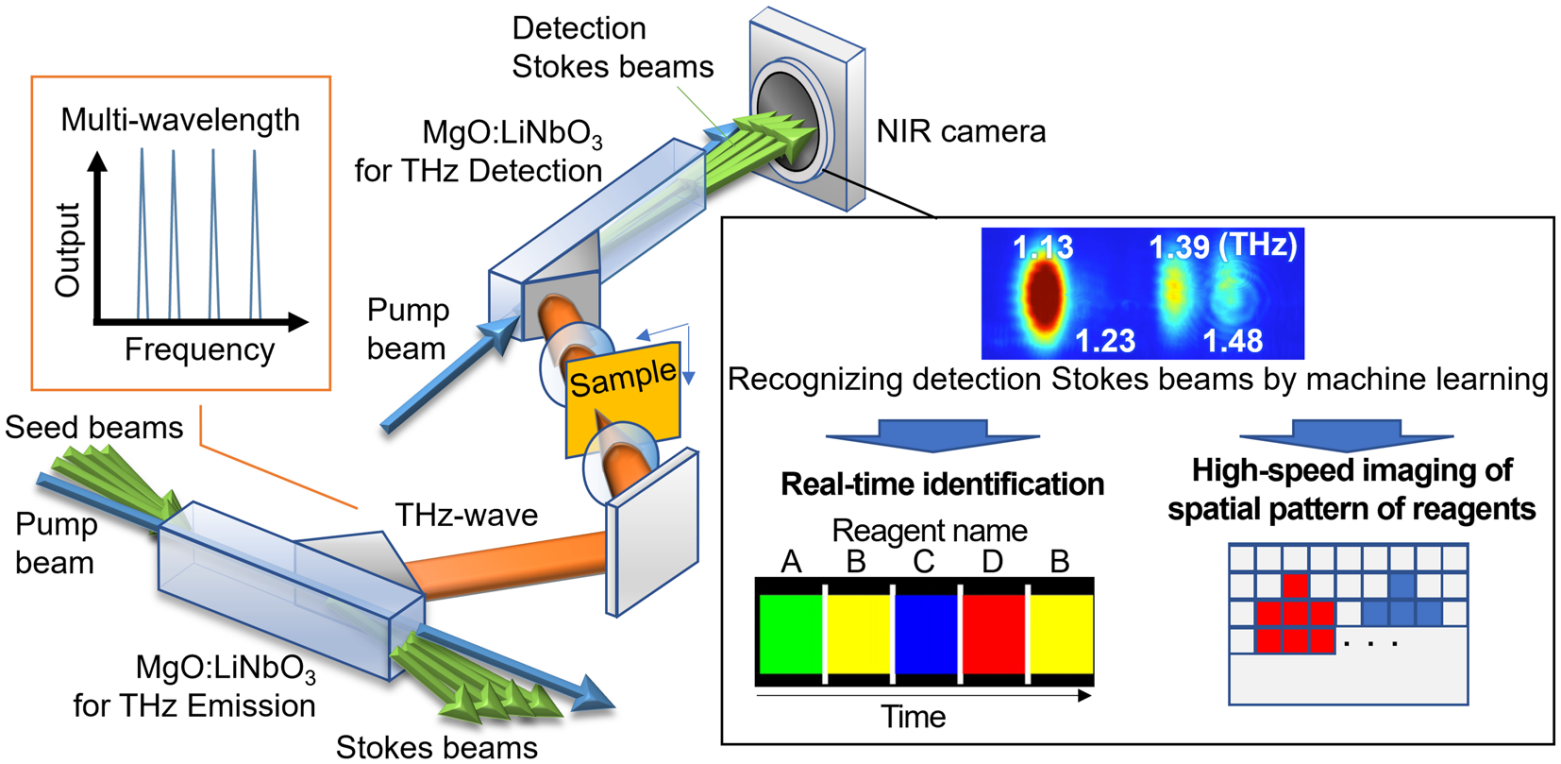
Real-time spectroscopic system that combines machine learning and multi-wavelength terahertz (THz) parametric generation/detection. We used a camera (FLIR, GS3-U3-41C6NIR-C) synchronized with the pump laser, with an exposure time of 30 ms and a frame rate of 20 Hz, for data acquisition. An overview of real-time spectroscopy and the high-speed imaging of reagents is shown on the right.

An overview of a THz spectroscopy system using an is-TPG is shown in Fig. 1. When the multi-wavelength seed beams are injected into the crystal with the pump beam, multi-wavelengths THz-waves are generated^15,16^. THz parametric detection^19^ is also possible via the reverse generation sequence, in which multi-THz-waves are used as seed beams, and NIR “detection Stokes beams” are generated and then captured by a camera. The generation angles of the detection Stokes beams are determined by the detected THz-waves according to the non-collinear phase-matching condition. Therefore, one-shot spectroscopy is achieved by converting the generation angles of detection Stokes beams into THz-wave frequencies. Since a frequency can be selected that avoids the absorption line of water vapor in multi-wavelength generation, purging with dry air is not performed.

**Figure 2:**
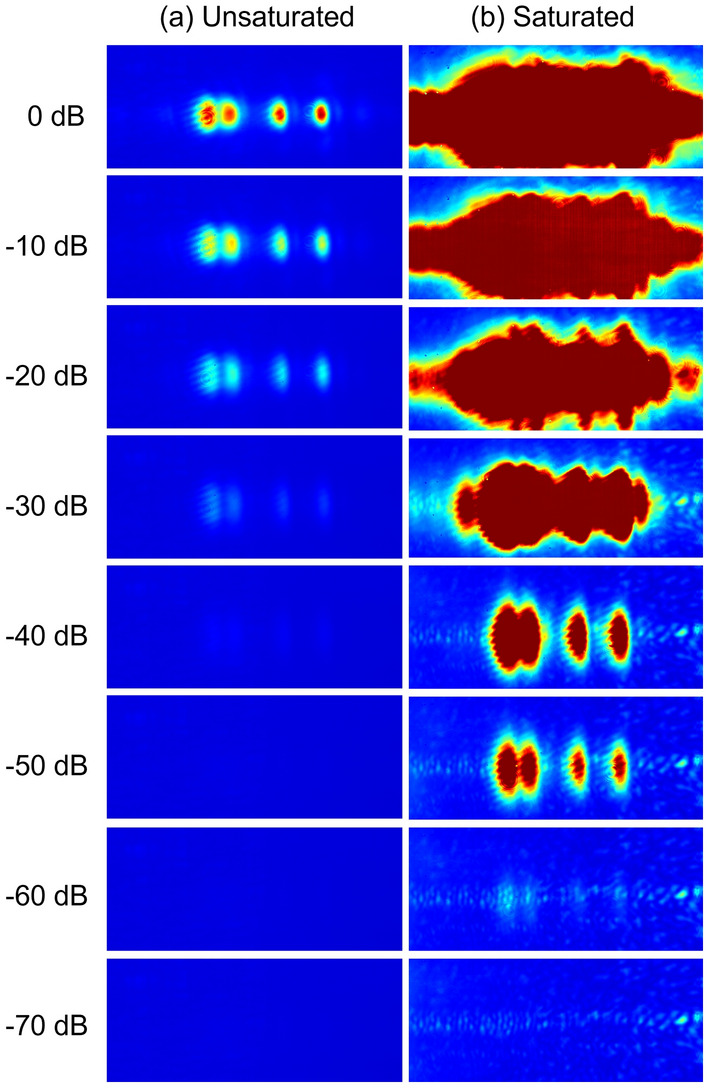
Evaluation of the dynamic range of a THz spectroscopy system. (**a**) Conventional method, in which the detection Stokes beam intensity at each wavelength is treated as a numerical value and only unsaturated beams can be injected. (**b**) With the new method, saturation is not an issue because image recognition is used, which allows for reagent identification over a wide dynamic range.

In this section, we describe the reagent identification method used in this study. For our previously reported THz spectrometer with a single wavelength is-TPG^16^, the output of the detection Stokes beam was measured by an NIR pyroelectric detector with a lock-in amplifier. Without adjusting the intensity by changing the neutral density filter, a wide dynamic range of nearly 80 dB (in terms of THz wave intensity) was obtained. On the other hand, in the multi-wavelength is-TPG, an NIR camera is used instead of an NIR pyroelectric detector to measure the “multi-wavelength detection Stokes beam” output with different generation angles. NIR cameras have been used to acquire the detection Stokes beam^15^; however, in these cases, the THz wave intensity is converted to a numerical value from the NIR camera image and then analyzed. Saturation is not tolerated, as no further numerical change can be obtained once the intensity saturates the camera. Thus, the dynamic range of the system in earlier studies was limited by the narrower dynamic range of the camera compared to the pyroelectric detector, which was about 40 dB, as shown in Fig. 2a. In addition, if images of the detection Stokes beam at adjacent wavelengths overlap when measuring the multi-wavelengths with a camera, the output becomes a mixture of information at different wavelengths and the spectrum cannot be measured accurately. Therefore, it is necessary to separate the beams so that they do not overlap, such that the wavelength spacing is wider.

In this research, the spectral information of the detection Stokes beam at each wavelength is recognized directly from the camera image by a convolutional neural network (CNN)^20^, which is a type of deep neural network, without converting the information into numerical values. Thus, features in each image are extracted by the CNN for learning and identification. If the multi-wavelength detection Stokes beam images obtained from the camera contain different features for each reagent, they can be identified by machine learning. Therefore, quantitative absorption information obtained as a numerical value is not necessary, and the overlapping of detection Stokes beams at adjacent wavelengths and saturation are not problematic. A THz spectroscopy system with a wide dynamic range capable of discriminating samples with low to high attenuation can be constructed by injecting the detection Stokes beam without any concerns about saturation. As shown in Fig. 2b, changes in the detection Stokes beam were captured from 0 dB (without shielding) to − 60 dB (through heavy shielding). Compared to the case without image recognition, the dynamic range was improved by > 20 dB.

**Figure 3:**
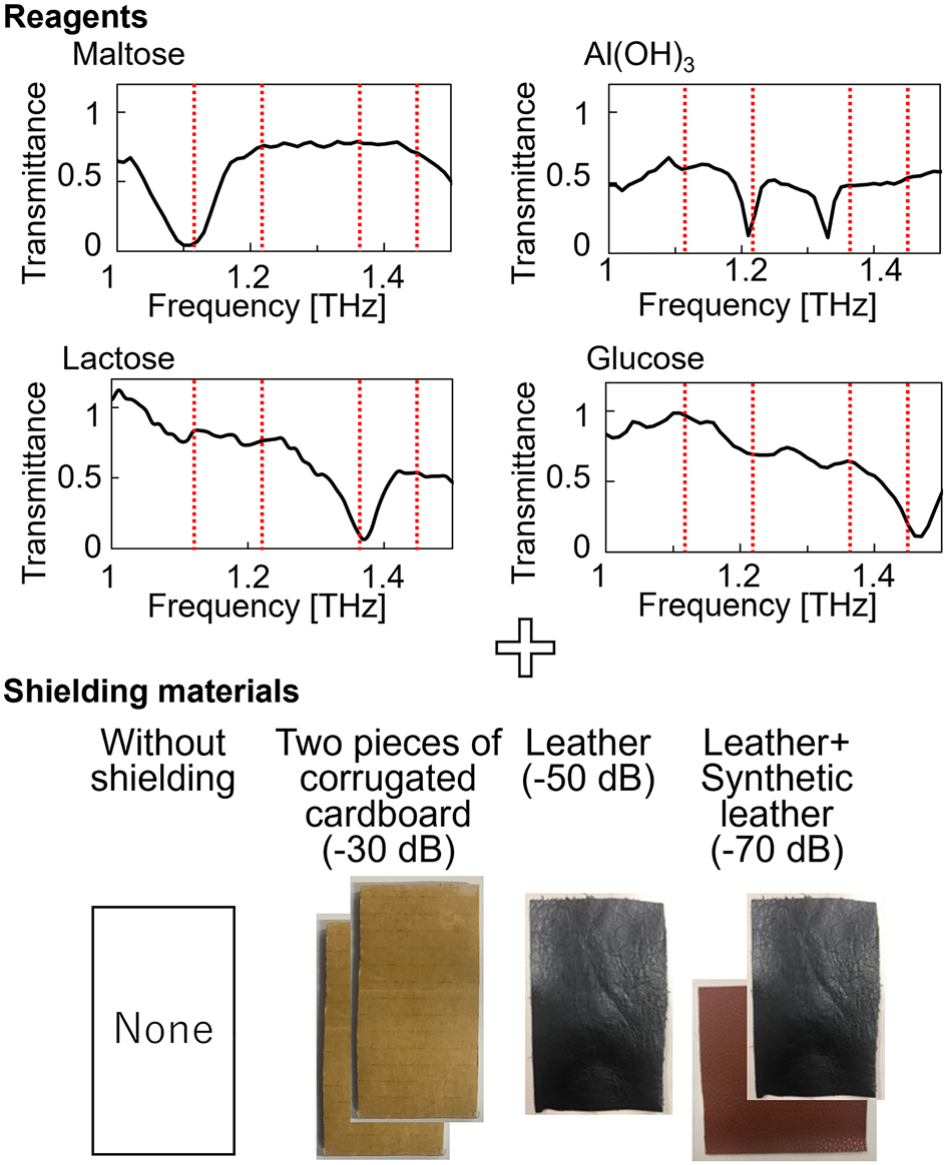
Absorption spectra of reagents and shielding used for the spectroscopy measurements, the results of which are shown in Fig. 4.

Real-time spectral discrimination was performed using the proposed system. Four reagents, maltose, Al(OH)_3_, lactose, and glucose, were used as the measurement samples; their spectra are shown in Fig. 3. Absorption spectra were measured using THz parametric detection after THz-wave output from the normal is-TPG was focused and transmitted through a sample placed at the focal point. A mixture of 75 mg of polyethylene powder and 75 mg of each reagent was compressed and pelletized in a hand press. Each sample was identified in the absence of shielding, through two sheets of cardboard (attenuation at 1.4 THz: − 30 dB), through natural leather (attenuation at 1.4 THz: − 50 dB), and through a layer of natural leather and a layer of synthetic leather (attenuation at 1.4 THz: − 60 dB). Four frequencies (1.12, 1.21, 1.37, and 1.44 THz; indicated by the red lines in Fig. 3) were used for the measurements based on the absorption spectrum of each reagent.

To identify reagents through any kind of shielding, it is necessary to train the CNN in advance with measurement data obtained under various attenuation rates. Therefore, a total of 1650 images of the detection Stokes beam, obtained during measurements of the reagents over various numbers of sheets of copy paper (0–21 with an increment of 3), were used as the training data. At this time, multiple pellets were prepared for each reagent, and the pellets for acquiring training data and those used for identification were not mixed. As shown in Fig. 2, the images of the detection Stokes beams were significantly different depending on the attenuation rate (low vs. high), and there was concern that the discrimination accuracy would be reduced by the shielding, even for spectroscopic measurements of the same reagent. Therefore, we prepared high- and low-attenuation classes for each reagent, and classified the targets into nine categories, including one in which no sample was inserted. Note that regardless of whether the sample is classified as high or low attenuation, the same reagent is shown. It means that the user is not aware that the classes are divided into high and low attenuation.

**Figure 4:**
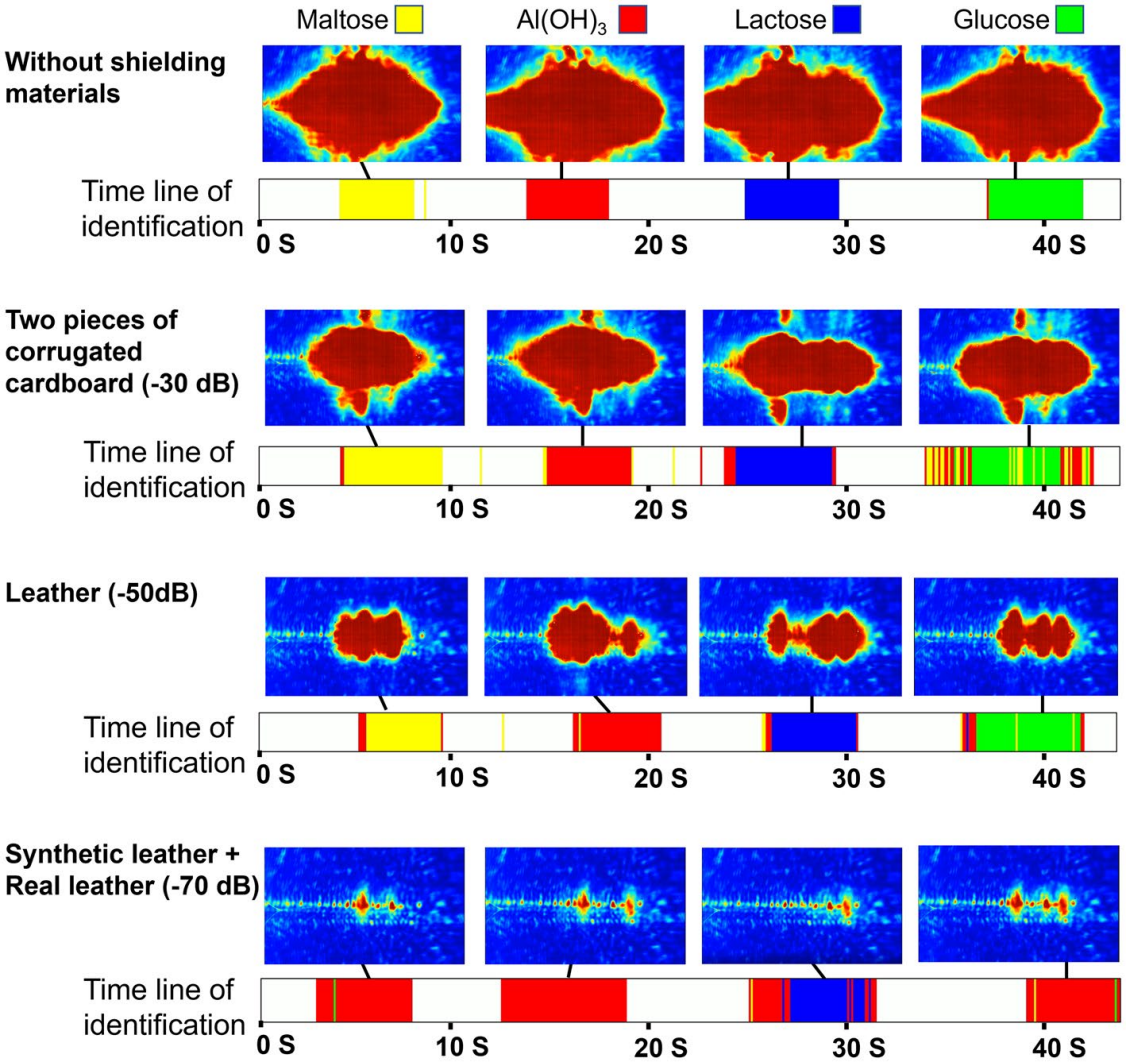
Real-time identification results obtained by machine learning (displayed in color) in chronological order, along with screenshots of the detection Stokes beam obtained when the reagent was inserted. We measured four reagents under 0 to − 70 dB attenuating shielding materials.

Figure 4 shows an image of the detection Stokes beam obtained when the reagent was inserted and the real-time identification results obtained by machine learning. The reason for the differences in background noise and measured beam between Figs. 2 and 4 is that the alignment was slightly different, even though the same experimental setup was used. The identification results obtained when the reagents were inserted in sequence are displayed in color, in chronological order. Errors are inevitable during reagent replacement due to disturbance of the detection Stokes beam. Although real-time identification was possible with high accuracy with attenuation up to − 50 dB, high-frequency components could not be detected through a shield with − 70 dB attenuation, and three of the four reagents were incorrectly identified as Al(OH)_3_. The system has a dynamic range of > 60 dB. Due to additional attenuation by the reagent itself, measurements were possible through shielding with attenuation of up to − 50 dB. When measuring through cardboard, which has an uneven or etalon structure, the identification error increases slightly, but high overall accuracy is obtained, indicating that our method is resistant to the effects of shielding. In comparison, at low attenuation, although the differences in detection Stokes images for each reagent were small due to saturation, the system was able to achieve highly accurate identification with almost no errors, indicating that machine learning is useful for qualitative identification of reagents.

In addition, this measurement is intended to be used in the field, and each sample is inserted into the optical path manually. Although the angles and positions of the samples were not precisely aligned, they were identified with high accuracy, indicating the high practicality of this system.

**Figure 5:**
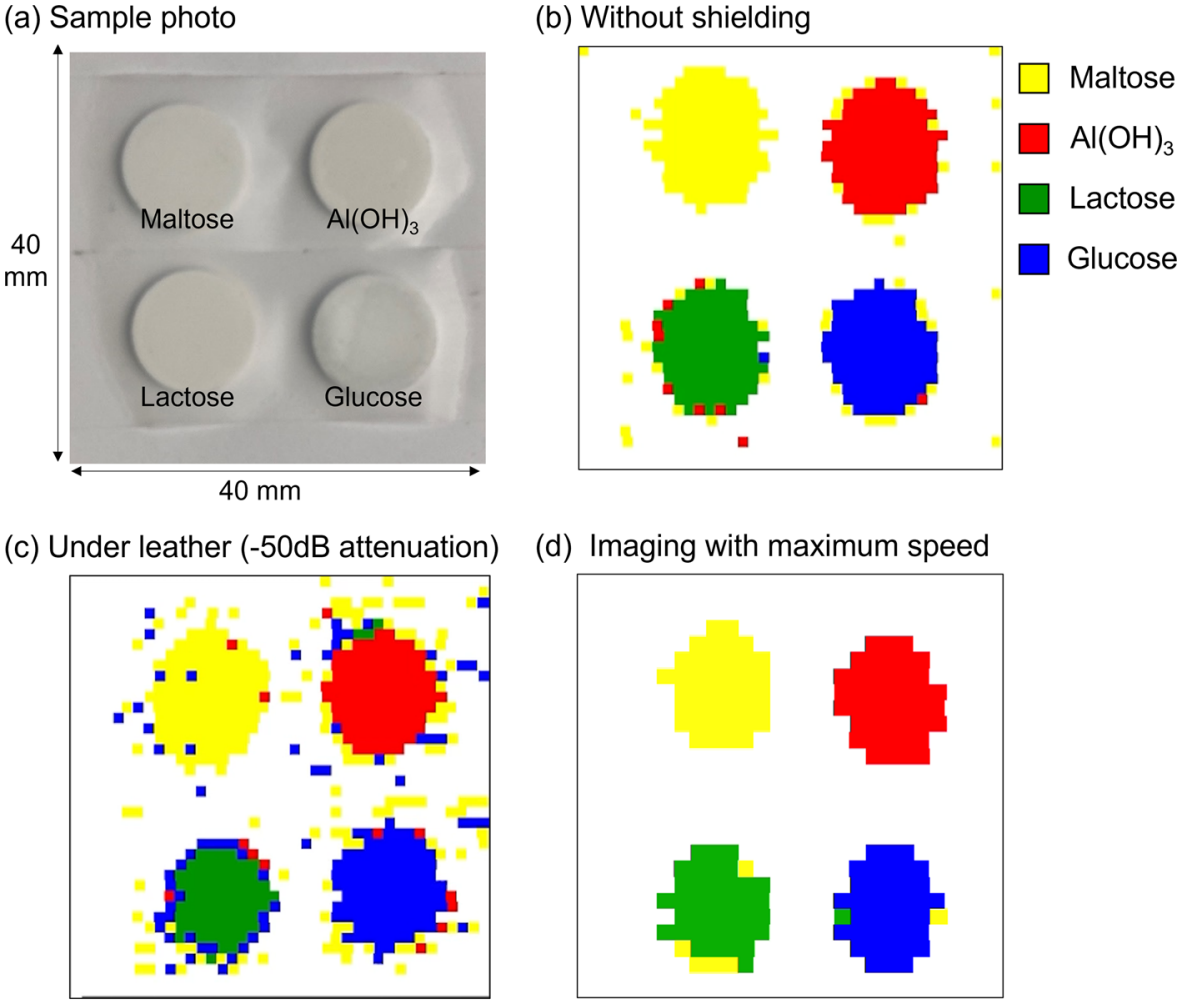
Spectroscopic imaging results. (**a**) Sample photo. (**b**) Four reagents were measured without shielding. (**c**) Four reagents measured through a − 50 dB attenuation shield. (**d**) Measurement with the maximum stage speed and reduced resolution. The spatial distribution of the reagents was visualized in < 1 min.

In conventional spectroscopic imaging using an is-TPG^2^, the stage is moved for each pixel, imaging is performed by averaging the values at each point, and the spatial distribution of the reagent is obtained through analysis. As the number of wavelengths increases, so too does the measurement time, such that several hours are required to obtain a single image. On the other hand, using the proposed system, information from multiple wavelengths can be obtained in one shot; thus, the measurement time does not increase with the number of wavelengths, and averaging is not necessary because machine learning enables instantaneous identification. The sample was continuously raster-scanned at a rate of about 26 mm/s using an automated stage, and the value of each pixel was identified simultaneously. Four kinds of pellets (50% reagent concentration) were arranged as shown in Fig. 5a, and spectroscopic imaging was performed (without shielding and through natural leather with − 50 dB attenuation). A 40 × 40 mm^2^ area was imaged with a spatial resolution of 1 mm. Not only the unshielded case (Fig. 5b) was imaged with high accuracy but also the − 50 dB attenuation case (Fig. 5c) was imaged with sufficient accuracy through the natural leather. The same system and training data were used to produce images under significantly different attenuation rates, and the results confirmed the wide dynamic range of the system. The images were acquired in 1 min 40 s, thereby reducing the measurement time to more than 1/100 compared to that of conventional systems^2^. Next, in an attempt to achieve even higher speed, we set the spatial resolution to 1.5 mm and the stage speed to its maximum of 30 mm/s; the measurement results are shown in Fig. 5d. Imaging was achieved in < 1 min, which represents a significant reduction in measurement time. Furthermore, the current measurement time is limited by the stage speed, so if a faster stage were introduced, measurement could be performed more quickly. In such a case, the repetition rate of the laser and frame rate of the camera need to be improved to obtain sufficient resolution.

**Figure 6:**
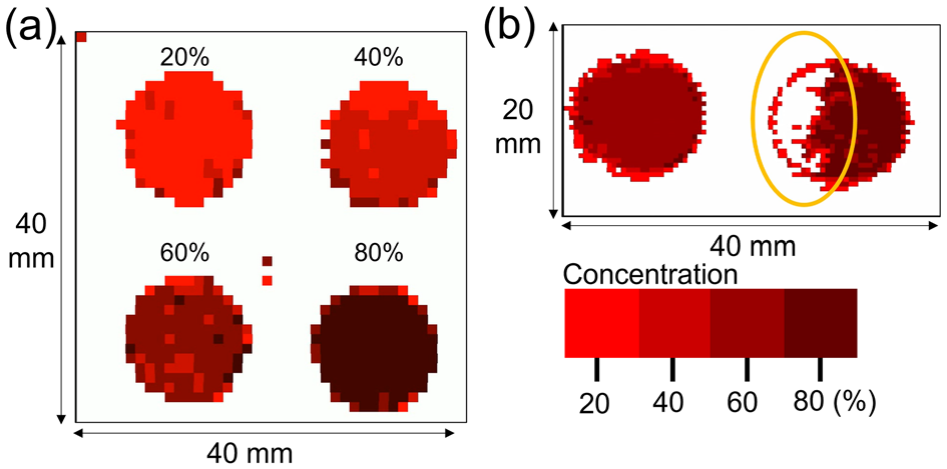
(**a**) Results of quantitative measurements for different reagent concentrations. (**b**) The uniformity of the reagents was evaluated using the same training data as in (**a**) (measurements were made with a spatial resolution of 0.5 mm).

Next, the concentration of the reagent was measured and the spatial distribution of the reagent within the pellet was imaged. While qualitative measurements such as those shown in Fig. 5 are sufficient for detecting illicit drugs and prescription drug errors, quantitative measurements are required for pharmaceutical factory lines and quality control of various reagents. Therefore, to measure the concentrations quantitatively, we divided the identification targets (classes) into different concentrations. Pellets of Al(OH)_3_ were prepared at concentrations of 20%, 40%, 60%, and 80% and used to train the machine learning model. The results of sample measurements obtained at different concentrations are shown in Fig. 6a. The system was able to discriminate among the concentrations, indicating the plausibility of quantitative measurement. Here, we measured pellets with a 20% difference in concentration; however, this system can distinguish differences of at least 5%. The system was also applied for the evaluation of pellet uniformity. Two samples were prepared with the same concentration of Al(OH)_3_, but one had a uniform distribution and the other had a non-uniform distribution. As shown in Fig. 6b, the concentration distribution can be visualized, which aids uniformity evaluation.

## Summary

Real-time reagent identification using a multi-wavelength is-TPG was achieved by introducing machine learning to recognize the detection Stokes beam image. The images can be identified even if they are saturated, i.e., they can be recognized even if there are slight differences. This means that highly accurate real-time identification can be achieved with the same setup and training data, from unobstructed to obstructed with − 50 dB attenuation. This system acquires training data at various attenuation rates, and it can discriminate signals through shielding at any attenuation rate as long as the signal is not buried in noise. Furthermore, by plotting the identification results obtained with this system as pixel values, the spatial distribution of reagents can be imaged more rapidly than before.

In conventional spectral imaging using an is-TPG, the measurement time increases with the number of wavelengths included. In addition, given that data processing and imaging results are displayed after all wavelengths have been measured, identification results cannot be obtained in real time. With our proposed method, the imaging time is reduced to several tens of seconds, and the results can be confirmed in real time without any post-processing. Given that both qualitative and quantitative measurements can be obtained with this system, we believe that it can be applied not only for mail and explosive testing applications, but also to prescription error testing in pharmacies, mixing ratio testing of reagents in chemical plants, and quality control in pharmaceutical factory lines.

## Method

### Terahertz generation and detection using is-TPG

When a high-power pump beam and seed beam are input to a MgO:LiNbO_3_ crystal, a Fourier transform-limited, narrow linewidth, high-brightness THz-wave is generated by parametric wavelength conversion^16,17^. At that time, wide tunability (0.4–5 THz) of the THz-wave can be achieved by controlling the wavelength of the seed beam and its incident angle such that the non-collinear phase-matching condition of the MgO:LiNbO_3_ crystal is satisfied. Using sub-nanosecond pump pulses from a microchip Nd:YAG laser, the is-TPG suppresses the induced stimulated Brillouin scattering, which is a competitive process^21^, thus allowing for high power generation with a peak power of ≥ 50 kW.

### Pump and seed laser sources for the is-TPG

As a pump source, we used a Nd:YAG MOPA system, which includes a microchip Nd:YAG laser and a Nd:YAG amplifier. It has 25 mJ pulse energy at a wavelength of 1064 nm, repetition rate of 50 Hz, and pulse duration of about 500-ps.

As a seed source, we used a combination of four external cavity laser diodes to generate multi-wavelength THz waves. The multi-wavelength seed beams were amplified to 300 mW using a semiconductor optical amplifier, and were then injected into the MgO:LiNbO_3_ crystal using an achromatic optical setup, such that the phase matching angles were automatically satisfied at all wavelengths.

### Machine learning method

A CNN, as a type of deep neural network, was used for image recognition. It has convolutional and pooling layers among its hidden layers. Convolution refers to the process of converting grid data (i.e., kernels) and numerical data for a partial image (of the same size as the kernel) into a single numerical value, by summing the products of each element. The local correlation is extracted by converting the data into small-grid numerical data, by gradually shifting the measurement window. Pooling is a method for reducing a large image while retaining the most important information, by dividing the image into small parts and extracting the maximum value from each part. By combining convolutional and pooling layers, it is possible to learn images efficiently.

For this study, the program was written in Python and the CNN framework was Keras. Our CNN model consists of three hidden layers and two fully connected layers. The first hidden layer comprises a convolutional layer with 30 filters (kernel size of filter: 3 × 3) and a pooling layer (kernel size of filter: 2 × 2). The second hidden layer comprises a convolutional layer with 20 filters (kernel size of filter: 3 × 3) and a pooling layer (kernel size of filter: 2 × 2). The third hidden layer comprises a convolutional layer with 10 filters (kernel size of filter: 3 × 3). For the activation function, ReLU is used for all hidden layers. The output from these hidden layers is converted into a 1-dimensional vector and then inputted into the fully connected layers. The first fully connected layer has 100 units and uses ReLU as the activation function. The second fully connected layer corresponds to a variable number of classes and uses a softmax function to output the class probability distribution. The model is compiled using cross-entropy as the loss function and Adam as the optimizer. In training the model, the batch size is set to 64, and the model is trained for a specified number of epochs (It was set to 300 in this study).

## Data Availability

Derived data supporting the findings of this study are available from the corresponding author K. M on request.
